# Process and feasibility of implementing guideline recommendations for the care of osteoarthritis in West Africa

**DOI:** 10.1136/bmjgh-2024-018714

**Published:** 2025-06-19

**Authors:** Opeyemi O Babatunde, Oladapo Adetunji, Ibidunni Alonge, Tolulope Owoyemi, Ebunoluwa Ayinmode, Adebimpe Ogunbanjo, Simon White, Adewale Adebajo Adebajo, Christian Mallen, Krysia Dziedzic

**Affiliations:** 1 School of Medicine, Keele University Faculty of Health, Keele, UK; 2 West African Institute for Applied Health Research, Ibadan, Nigeria; 3 University of Ibadan, Ibadan, Nigeria; 4 Lagos state health service, Lagos, Nigeria; 5 Keele University, Newcastle-under-Lyme, UK; 6 The University of Sheffield Faculty of Medicine Dentistry and Health, Sheffield, UK

**Keywords:** Global Health, Health services research, Infections, diseases, disorders, injuries, Treatment

## Abstract

**Objectives:**

To assess the feasibility of a guideline-informed model of care for osteoarthritis in primary healthcare and community pharmacy settings in the West African context.

**Methods:**

The 4-phase mixed-methods programme of research undertaken in Southwest Nigeria, West Africa. Phases 1–2 involved contextual adaptation of guideline-informed care**—**Joint Implementation of Guidelines for OSteoArthritis in West-Africa (JIGSAW-A): (1) focus groups (n=4) with patients, community pharmacists and healthcare professionals (HCPs) to identify patient preferences and support needs of HCPs; (2) stakeholders resource contextualisation/codesign (ie, osteoarthritis guidebook in local languages, HCPs training/support package). Iterative codesign workshops (n=3) using participatory approaches, model osteoarthritis consultation simulations and consensus agreement.

Phase 3: following training and a 12-week pilot implementation period, patient-reported quality of care was assessed by the OsteoArthritis Quality Indicator questionnaire (modified 9-item scale 0%–100%, 100%=best), and implementation of the JIGSAW-A model of care was evaluated using the Reach-Effectiveness-Adoption-Implementation-Maintenance framework. Patient and HCP interviews explored barriers and facilitators, usefulness and acceptability. In phase 4, recommendations for further scale-up and wider implementation of integrated osteoarthritis care were specified.

**Results:**

Phases 1–2 highlight the burden and impact of everyday living with joint pain and misinformation which affects help-seeking. Participants expressed the need for a broad information and education campaign and access to self-management support, which informed iterative contextualisation of osteoarthritis care and patient information resources used to support pilot implementation in phase 3.

Over 12 weeks, 12 HCPs (community pharmacies, physiotherapists and doctors) were involved in evaluation across nine sites. Of 369 patient consultations that were reported, high rates of quality indicator achievement were found for self-management advice (97%), topical analgesic use (89%) and exercise recommendations (87%). Compliance with full patient assessment in line with the protocol was poor (17%).

**Conclusions:**

We found that evidence-based care for osteoarthritis, involving community pharmacies (as a usual first point of call) and other primary care clinicians, is feasible and may improve aspects of care in low-resource settings. Further research is needed to ascertain long-term sustainability and cost-effectiveness.

**WHAT IS ALREADY KNOWN ON THIS TOPIC**

- Africa contributes up to 21% to the increasing prevalence of osteoarthritis globally.
- There are evidence-based resources that can potentially support care and patient self-management in real world settings, but these are underpinned by research largely from North America and Europe and have yet to be contextualised for use in West Africa and other low-resource settings.

**WHAT THIS STUDY ADDS**

- Evidence-based care for osteoarthritis is feasible and can improve quality of care in low-resource settings.
- Being a usual first point of call for many patients seeking symptom relief, community pharmacies have a critical role in osteoarthritis management through brief education/advice and signposting to other appropriate treatments (eg, physiotherapy-led exercises).
- People living with osteoarthritis in low-resource health settings (eg, Nigeria, West Africa) increasingly value evidence-based health information and advice for managing their conditions.

**HOW THIS STUDY MIGHT AFFECT RESEARCH, PRACTICE OR POLICY**

- There is a need to establish further the clinical and cost-effectiveness of guideline-informed care for osteoarthritis across low-resource settings.
- With the increasing prevalence of osteoarthritis and the potential effect on productivity, long-term health system infrastructures to support people to self-manage will likely yield a good return on health and socioeconomic investment, particularly in Africa, where a large proportion of people living with osteoarthritis are women and are of working age.

## Introduction

 Africa contributes significantly to the increasing global prevalence (>21%), unmet needs and treatment burden for osteoarthritis.^1^,^3^ Characterised by pain, disability and impaired quality of life, osteoarthritis is a serious joint disease with direct and indirect costs of treatment and work-related losses leading to considerable economic burden and widening health and social inequity.^1 4 5^ Significant investments have led to the development of high-quality clinical guidelines^6^ and evidence-based resources^7^ that can potentially support care and patient self-management in primary care and community settings, yet implementation of these recommendations in practice continues to lag in real-world settings.^8^ Furthermore, current clinical guidelines and evidence-based resources are largely underpinned by research from North America and Europe and have yet to be contextualised for use in West Africa and other low-resource settings.

Due to ‘cultural conventions/misconceptions’ about their conditions, out-of-pocket healthcare expenses and ease of access, people living with osteoarthritis in Nigeria and most West African countries commonly self-medicate or seek help from community drug retail outlets and pharmacies to relieve pain before and alongside seeking formal advice in clinics and hospitals.^9 10^ Despite the high osteoarthritis prevalence and symptom burden, and known use of community pharmacies by those living with chronic pain,^1^,^310^ no research has examined the role of community pharmacists and their support needs for managing osteoarthritis in West Africa.

While there is currently no known cure for osteoarthritis, structured management programmes/care packages which include core treatments such as patient education, exercise and weight management have been recommended with the goal of reducing symptoms and improving function.^11^ To our knowledge, there is no report yet of a structured osteoarthritis management care programme with core guideline recommendations contextualised and implemented in West African settings. Based on previously successful osteoarthritis research and implementation programmes in Europe, that is, the Joint Implementation of Guidelines for OSteoArthritis in West-Europe (JIGSAW-E, https://jigsaw-e.com/), this study (Joint Implementation of Guidelines for OSteoArthritis in West-Africa (JIGSAW-A)) was a pilot implementation project conducted in Nigeria as a starting point to addressing research and practice gaps in improving quality care for osteoarthritis in West Africa. The overarching aim of JIGSAW-A was to improve the availability of evidence-based care for people with osteoarthritis by empowering patients with high-quality, accessible information and supporting pharmacists and other healthcare professionals (HCPs) to deliver osteoarthritis care within an integrated primary healthcare system, in line with core guideline recommendations. Across four iterative study phases, specific objectives were to:

1. Understand local context-specific patient information needs; community-based pharmacies support needs to deliver evidence-based care by exploring patient and HCPs’ views and experiences regarding osteoarthritis care in Nigeria.
2. Culturally adapt, contextualise and refine a previously developed (ie, existing JIGSAW-E tools) osteoarthritis patient guidebook to aid patient education and advice as well as HCPs’ care support package.
3. Evaluate the acceptability, usability and impact of the JIGSAW-A approach on osteoarthritis care among patients and HCPs using JIGSAW-A resources in Nigeria.
4. Develop knowledge mobilisation strategies/recommendations for the wider implementation of conceptualised JIGSAW-A resources and evidence-based care for osteoarthritis in West Africa.

This paper focuses on the process, feasibility evaluations of the JIGSAW-A pilot in Nigeria and recommendations for wider implementation (objectives 3 and 4). Specifically, we sought to understand HCPs’ and patients’ perspectives of the perceived effect of the care they delivered (or received) as informed by JIGSAW-A and explore whether the results are likely to be generalisable, scalable and sustainable by exploring stakeholders’ experiences of the JIGSAW-A model of care and its perceived impact.

## Methods

### Study design and conceptual framework

**Figure 1:**
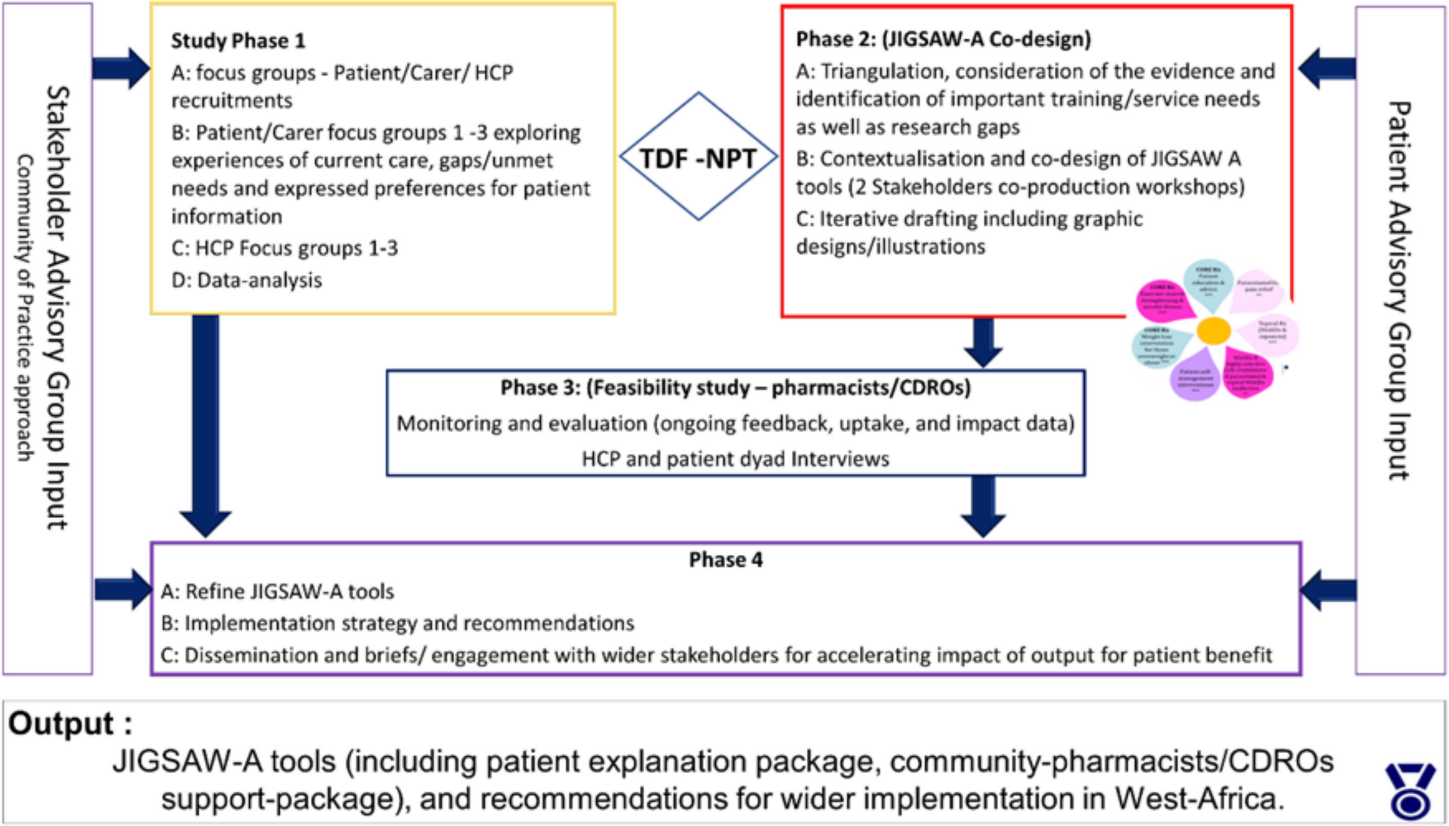
Overview of the JIGSAW-A project. HCP : healthcare professional; CDRO: Community drug retail outlets, JIGSAW-A : Joint Implementation of Guidelines for OSteoArthritis in West-Africa.

JIGSAW-A is a mixed methods pilot implementation project (figure 1) aimed at improving the quality and value of care in primary care and community settings and is reported based on the Standards for Quality Improvement Reporting Excellence (SQUIRE) reporting guidelines.^12^ The procedures followed were in accordance with the ethical standards of the responsible committee on human experimentation (institutional and national) and with the Helsinki Declaration of 1975, as revised in 2000. Ethical approval was sought and obtained from the Research Ethics Committee of the University of Ibadan, Nigeria (reference: UI/SSHEC/2022/0023), and Keele University (KU0420).

This project aligns with the development and feasibility stages of the Medical Research Council’s guide for developing and evaluating complex interventions.^13^ It was informed by the Theoretical Domains Framework (TDF),^14^ and the Reach-Effectiveness-Adoption-Implementation-Maintenance (RE-AIM) framework^15^ was used to evaluate implementation outcomes. The RE-AIM was used to enhance understanding of how best to implement context-specific integrated care for osteoarthritis, involving community pharmacies and other HCPs.

Detailed methods and findings from phases 1 and 2 have been reported elsewhere^16 17^ and are briefly summarised below:

#### Phase 1

Five focus group discussions were held. Three included people living with osteoarthritis (n=30, age range 45–90 years)^16^ who described their experiences of living with osteoarthritis as emotionally, physically and socioeconomically challenging. Participants expressed the need for a broad information and joint health education campaign and access to appropriate health professionals (especially physiotherapists) for providing support, guidance and assistance with self-management.

**Figure 2:**
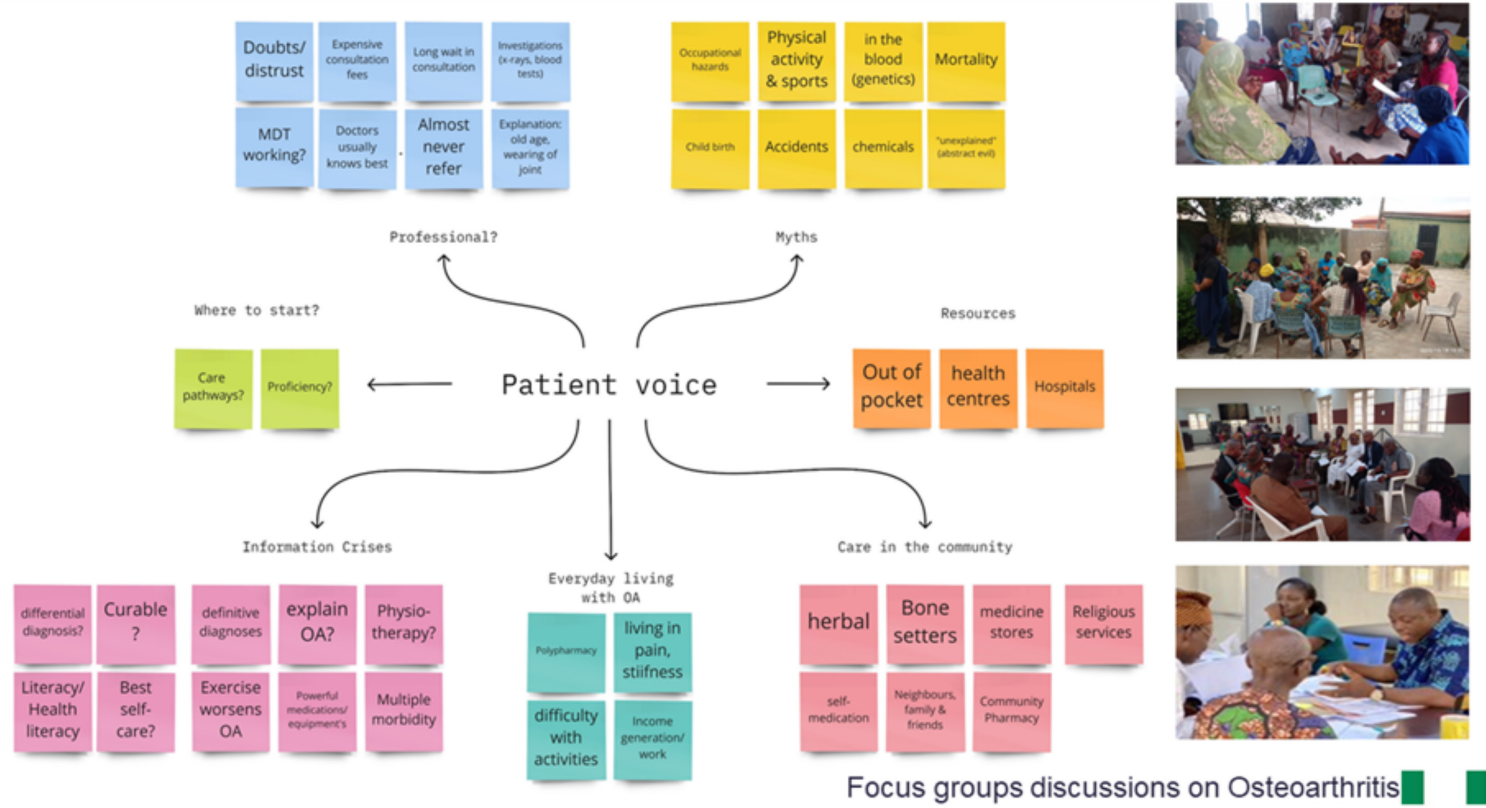
JIGSAW-A phase 1 summary of findings. MDT; Multidisciplinary Team

The two focus group discussions were conducted with 22 HCPs (including community-based pharmacy teams, physiotherapists, nurses and doctors), with 5–39 years of practice (mean=21.5). Discussions confirmed community pharmacies and drug retail outlets as the usual first point of call for most patients and participants expressed the need for professional education and context-sensitive national guidelines, codeveloped and agreed to inform the care of patients with osteoarthritis and joint pain. Summary of findings from fact-finding in phase 1 which informed contextualisation and pilot implementation of JIGSAW-A is presented in figure 2.

#### Phase 2

Stakeholder contextualisation and codesign of JIGSAW-A resources followed an iterative design involving three stakeholder codesign workshops, which included osteoarthritis consultation simulations and were complemented with ongoing feedback throughout. Stakeholders (n=45), who were recruited through known networks and broad advertisements, included patients/carers, pharmacists and pharmacy technicians, physiotherapists, doctors, nurses, rheumatologists, orthopaedic surgeons, representatives of professional bodies (eg, Nigerian Society of Physiotherapists, Nigerian Association of Community Pharmacies), local voluntary sector organisations, osteoarthritis researchers and implementation and communication experts. They formed a community of practice (CoP) which contributed to the JIGSAW-A tool contextualisation phase and strategic recommendations in phase 4 (see below). The resulting JIGSAW-A resources used in pilot implementation consisted of:

1. Contextualised training programme for HCPs,
2. Patient information resources (short and long versions of osteoarthritis guidebooks in local languages),
3. Quality care indicator templates in local languages to assess care given,
4. JIGSAW-A consultation log to support assessment and management and
5. Model care map and algorithm.

#### Phase 3: feasibility study of JIGSAW-A

##### JIGSAW-A model of care

The JIGSAW-A care was conceptualised by the research team together with stakeholders as structured care for patients with osteoarthritis, many of whom start their journey of care in community pharmacies. Supported by an algorithm (irrespective of starting points, see online supplemental appendix 1), HCPs were instructed to deprioritise the need for X-ray investigations where clinical diagnosis confirms osteoarthritis and give education and information on osteoarthritis as a long-term condition (LTC). They were also instructed to use guideline-recommended treatment approaches, provide pharmacological treatment/deprescribe when appropriate, advise on exercise and suggest referral to physiotherapy where feasible.

### Training of health professionals

A core strategy for the implementation of the JIGSAW-A model of care was the training of multidisciplinary HCPs. Participating sites and associated HCPs were recruited from participants in phase 1, existing networks of the study team and advertisement via a professional association. Consenting sites were selected to represent the professional role/context of work (community pharmacies—70%), size and location of practice. HCPS attended a 3-hour JIGSAW-A interactive workshop and online training informed by current evidence and guideline recommendations for managing osteoarthritis (six modules using participatory approaches including vignettes to encourage interactions among participants and deepen engagement with learning). An outline of the training is presented in the online supplemental appendix 1. A second 1-hour ‘booster’ training session (4–5 weeks later, 11 HCPs working across participating sites) was conducted to confirm the use of the JIGSAW-A guideline-informed model of care, reinforce learning, address early implementation challenges and clarify the evaluation process.

### Management protocol and feasibility evaluations in participating sites

Post-training, JIGSAW-A resources and study packs (containing a participant interview invitation letter and a JIGSAW-A study information sheet) were distributed to consenting sites (community pharmacists and physiotherapists). Training certificates and slide presentations, together with supplementary explanatory notes, were provided to the trained professionals for reference (as needed). Fortnightly site visits/telephone calls were then conducted to offer technical support as needed to participating sites.

The management protocol included patient assessment, education and advice on self-management using the JIGSAW-A guidebook, pain medicines review and/or referral where necessary using the care map/algorithm. Additionally, an indicative exercise therapy protocol which could be used was outlined. Trained community pharmacists (n=7), a family physician (1) and physiotherapists (2) completed the training (including the booster sessions) and delivered the intervention. Key targets were to empower patients with understandable evidence-based information, thereby enhancing self-management; reduce use of unnecessary imaging for diagnosis and assessment and de-emphasise routine use of pain medicines in the hope of relieving their joint pain.

The feasibility of guideline-informed JIGSAW-A care for people living with osteoarthritis was then assessed in community pharmacies and health centres in Southwest Nigeria (Oyo and Lagos State). Evaluation involved analysis of consultation logs and review of quality-of-care indicator questionnaires from each site. Using topic guides, developed by the research team based on previous literature and with input from public contributors, semistructured interviews were held with HCPs who delivered the intervention and consenting patients (approximately two per site). Patient participant recruitment was pragmatic via the HCPs to involve approximately two patients (per site) who consulted for osteoarthritis/joint pain during the 3 months of pilot implementation leading up to the evaluation. To be eligible, willing participants had to be adults aged 45 years old or over with joint pain likely to be attributable to osteoarthritis. Vulnerable individuals (eg, people in a palliative phase of care, or people with unstable mental health) and those unable to provide informed consent were excluded.

The interviews were used to gain more detailed information about the usability, accessibility and usefulness of JIGSAW-A resources. The interviews explored participants’ experiences of care (patients) or management of patients with osteoarthritis (health professionals), including priorities for delivering care for osteoarthritis as an LTC and perceived barriers and facilitators related to the use of the JIGSAW-A tool for delivering care. Open questions at the end of the interviews allowed participants to discuss anything else they considered to be relevant and important and further exploration of any unanticipated issues raised by participants during the interview. The interviews lasted no more than 1 hour and were held face-to-face in line with established ethical principles and audio recorded. Verbatim transcriptions of interviews were checked for accuracy against the audio recording and fully anonymised. All participants provided written and verbal informed consent.

### Analysis

Descriptive analysis of consultation logs data was used to capture indicative practice per participating site over the 3-month period. The patient-reported quality of osteoarthritis care as assessed with the Osteoarthritis Quality Indicator questionnaire reflects core guideline recommendations. Patient participants were instructed to consider the treatment, information or advice they had received from the HCP (at respective site) within the 3-month period. Items in the questionnaire had ‘Yes’/‘No’ and ‘Not applicable’/‘Don’t remember’ as the response options. Each quality indicator item was considered achieved if the patient had checked ‘Yes’ for that item. Achievement of the quality indicator item was calculated as the total number of indicator items achieved divided by the number of eligible items for each patient (in percentage).

A thematic analysis of interview transcripts was performed to identify relevant themes broadly within the structures and domains of the RE-AIM framework. Coding and analyses were performed using Nvivo (NVivo qualitative software (V.12). To minimise the risk of researcher bias, five out of 22 interviews were randomly selected and independently coded and discussed by two researchers (OOB and TO), and following consensus, coding was continued by one researcher (TO). Descriptive analysis was followed by an interpretive analysis, through several cycles. After multiple readings, descriptive codes and categories were generated from the transcript data. These were clustered and reclustered to inductively identify themes and subthemes, which were then mapped to the RE-AIM framework (by the core study team members OOB, OA and IA). The themes, feedback and notes from site visits were examined and presented to the wider study team and public contributor for interpretation of findings, focussing on what did and did not work, lessons learnt and areas for refinement.

### Public involvement and engagement

The project recruited five people to receive training and provide patient and public involvement and engagement perspectives to the research by sustained contributions to key aspects, including the design, conduct, interpretation and dissemination of findings. Meetings were held bimonthly throughout the research process. Public contributors attended and made contributions to discussions in study steering group meetings (×3). As needed (total=4 times), public contributors also attended research team meetings to provide advice on recruitment strategies, contextualised resources and implementation activities. Specifically, two male public contributors (aged ≥65 years with lived experience of osteoarthritis of hip and knee, >5 years) were involved in site monitoring visits with researchers, broaching conversations with practitioners about the importance of the JIGSAW-A study and personal testimonies of the impact of the JIGSAW-A guidebook on own approach to self-management (see quote below, shared with permission).

It was a busy time, and they were being selective in the kind of patient they gave the guidebook to. I know they can’t give anybody anyway as there is not enough to go round. But after I spoke to him as a patient representative, while I was there, he had to bring out the file and began to fill in the consultation log. He said, I'm going to administer the questionnaire also on other persons. I understand the impact better now …I believe in your experiences

Public contributors also gave talks at community engagement events (road shows), which helped to raise awareness and share information about osteoarthritis and the JIGSAW-A model of care.

## Results

Of 15 sites that initially expressed interest in taking part in the feasibility study, eight community pharmacies and two health centres with at least one JIGSAW-A trained community pharmacist /HCP provided consent to participate and were enrolled. One community pharmacy later dropped out, citing high workload, insufficient time commitment to deliver care in line with JIGSAW-A and lack of incentives. In total, 19 semistructured interviews were completed (eight HCPs—one community nurse, one physiotherapist, six community pharmacists and 11 patients with osteoarthritis) across the nine participating sites. A description of themes mapped to domains of the RE-AIM framework is provided below and summarised in online supplemental table 1.

### Reach

**Figure 3:**
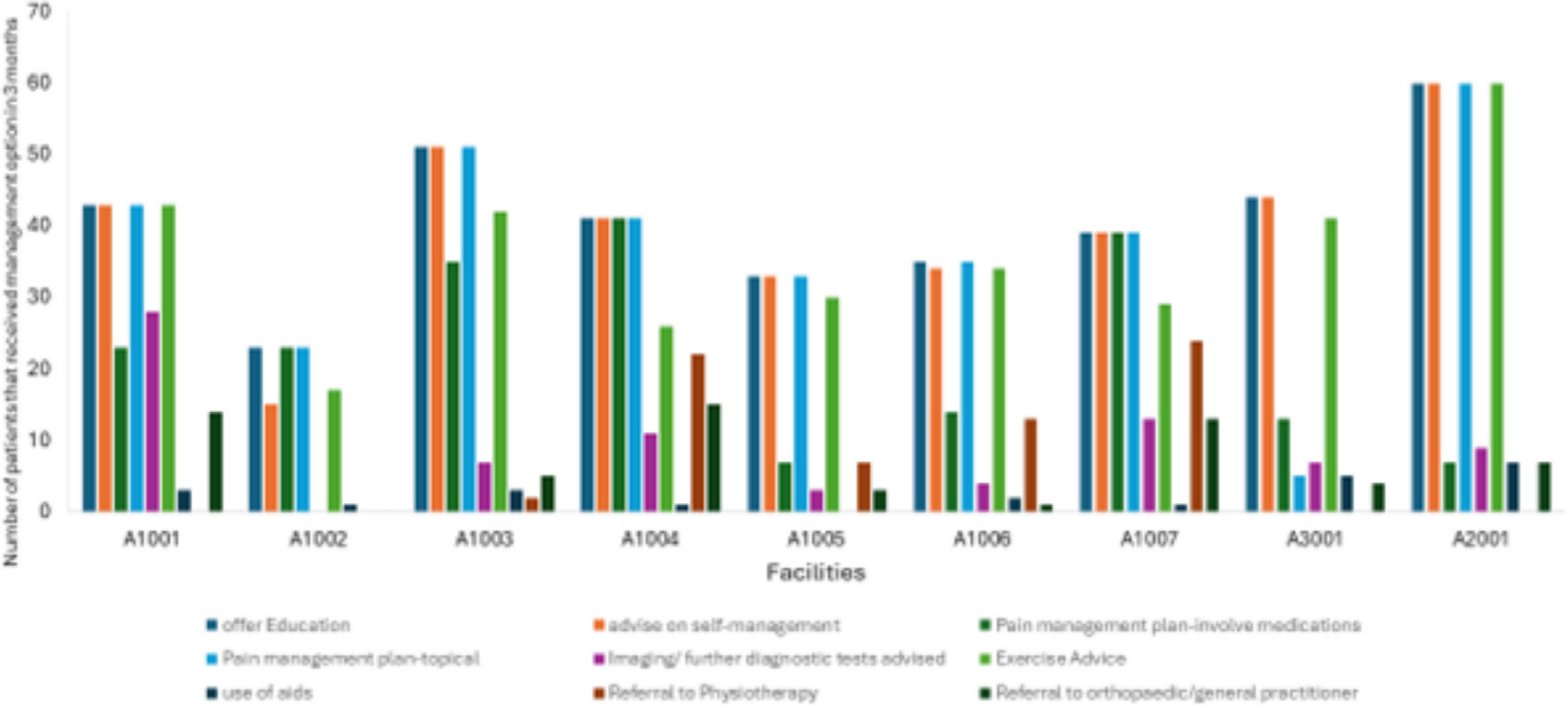
JIGSAW-A pilot sites management options offered across each facility over 3 months.

Over the course of 3 months rolling using JIGSAW-A resources, sites reported an average caseload of 10 (range 4–13) osteoarthritis patients per day. In total, 976 patients were reportedly provided with JIGSAW-A patient guidebooks across sites over the course of 3 months. Review and analysis of practice logs and across participating sites showed 369 patients were provided consultation in line with the JIGSAW-A model of care (figure 3 and online supplemental table 2)

*HCPs* who were interviewed all highlighted that JIGSAW-A training enhanced their knowledge of current evidence on osteoarthritis and guideline recommendations. They reported being open to aligning their practice to evidence-informed care for osteoarthritis:

At least I got a different perspective asides just prescribing medications. An emphasis on exercise, diet and all these support resources has been helpful and I'm sure it will be helpful to other professionals too. HCP8

I would say yes it impacted our practice because it now made me know that there’s actually more I can do in joint management, there’s so much more than giving pain killers*. HCP1*

However, some HCPs were selective in giving out the JIGSAW-A guidebooks:

It’s not many and we're not able to give it out to all… They could finish within a week! HCP 4.

let me be honest with you… because I saw that it took a lot of money to print those things. So it won't be fair if I give it to some person who will just abandon it. ….Africans don't read, they like pictures and funny videos that would illustrate what to do. They don't like lengthy things. So if it can be, make it into a fancy card or probably an interactive version so that everyone would be able to use HCP 9.

### Experience of the effectiveness of the JIGSAW-A model of care

The JIGSAW-A model of care was perceived to be largely beneficial. HCPs explained that JIGSAW-A resources expanded their knowledge of current evidence and increased their awareness of the critical role of information giving as well as best practices for the management of osteoarthritis.

I am more conscious of the fact that we, you don't need X-ray to diagnose osteoarthritis HCP1.

To reassure all my patients about their pain and then it helps to reduce their worry, it helps to reduce their anxiety and they focus on the exercises I show them to do HCP6.

Given the short term between implementation and evaluation, not all patients reported relief of symptoms, but many expressed being empowered with information and the need to reduce over-dependence on pain medicines.

There is nothing compared to a person doing exercise, because I observed changes whenever I do the physio exercise. I didn't buy any drug nor did I go to their place [referring to prior physician-practice not connected to JIGSAW-A] ever since then because they confirmed that it is only operation I can do and the chances of the operation is 50/50 P9.

They gave me chance to express myself. I was informed on how to take good care of myself and I’m practicing physical exercises daily. The instructions given in the guidebook are useful. The joint is not paining me as much again and I am satisfied with the explanation given me. I pray for them P2.

### Adoption

In relation to actual adoption, trained HCPs across sites adopted the JIGSAW-A model of care in their practice. However, they felt effective adoption requires system-wide training, shared understanding and cooperation among interprofessional networks. They explained that JIGSAW-A resources should be primarily adopted by the health boards and professional organisations such that managing patients using the JIGSAW-A model of care would not be an individual HCP style but a health system-wide approach where multidisciplinary professions involved in osteoarthritis care (eg, physicians, physiotherapists, nurses and orthopaedic surgeons) use the JIGSAW-A consistently as appropriate for each patient.

So I would say that here, if JIGSAW-A can bring all of us together, why not? For myself I would say that yes, it opened my eyes so that I can actually work better with physiotherapists HCP6.

The JIGSAW-A resources also received recommendations and endorsements from two professional associations (community pharmacies and physiotherapy).

In relation to the adoption process*,* many of the trained HCPs alluded to sharing information and resources from JIGSAW-A training with members of their team. Though the extent to which the training was cascaded down and adopted by other practitioners was untested and is therefore unknown. Furthermore, HCPs identified several factors that impacted the adoption of the JIGSAW-A model of care. In particular, they reported being short of time due to the high patient caseload. In addition, the shift in priorities (eg, away from purely pharmacological management of pain and there being less emphasis on the use of imaging in assessment and diagnosis) in the JIGSAW-A approach introduced uncertainties/incompatibility with current workflows for most HCPs and also raised concerns about income generation for community pharmacies.

sincerely I have not had time to follow through with them and the reason is very simple. We have four branches like this, so I also have other things that I am doing and they are kind of overwhelming. HCP9

Furthermore, to adopt self-management, including the use of the JIGSAW-A guidebooks, many patients required additional help: “*My children help me read the book to my hearing” P2.*

### Implementation fidelity

Figure 3 presents trends in use of the JIGSAW-A approach to management of osteoarthritis per site over the course of a 3-month implementation period. There was variation in implementation across sites, with high rates of quality indicator achievement found for advice on self-management (97%), use of topical analgesics (89%) and recommendations for exercise (87%). Only sites with physiotherapists (n=2) undertook full patient assessment in line with the protocol. Community pharmacies routinely supplied and/or prescribed topical gels, which may have been an indirect compensation for the shift from deprescription of routine pain medicines that was emphasised during training.

Most HCPs provided the quality indicator questionnaire to patients after consultations but stated that this method resulted in patients taking questionnaires home without returning them. In other instances, feedback on care received was requested/provided verbally without the use of the quality-of-care indicator questionnaire, which may have resulted in some patients providing socially desirable answers.

Some community pharmacists explained that the JIGSAW-A resources could not be used as per protocol for all potentially eligible patients, given the time and resources involved in providing detailed consultations, as well as patient expectations (eg, of expecting a curative treatment) and affordability of other approaches to care (eg, physiotherapy). This was corroborated by patients:

I thought there were other things they would give me asides the cream. My expectation was that once I get drugs and use them the pain will go P3.

Yes, I did see changes but not much. What I want is for my knee not to pain me ever again. P6

### Maintenance

Most of the HCPs reported still using elements of the JIGSAW-A model of care and resources at the time of the evaluation, and most of them expressed a desire to continue using the approach. However, all said they had run out of the JIGSAW-A guidebooks (the patient explanation package), which appeared to have been a major incentive to using the JIGSAW-A model of care. The main expressed needs of patient participants, which were also reinforced by health professionals, were for more engaging resources in visual/audio formats and patient support group networks to promote guided self-management and therapeutic exercises in the longer term.

I don’t know if JIGSAW Africa, your organisation can do it for us. You will gather us together and train us again at a convenient time and you will bring a physiotherapist on that day to check us so that we can enjoy you for that day. That will not be too expensive for us, that will also help us with this situation *P1.*

The feedback has been good, …but some of them still find it difficult to read. Not because they cannot read, actually, they still ask that you should put them through. But for people that read it, digest it very well, they still come back to say thank you and that I even gave this one you gave me to another person, which is very, very good. HCP 8

Participants also emphasised the need for wider campaigns to discourage over-dependence on medicines for the management of osteoarthritis.

For such program, we will know the days we have physiotherapy sessions and the token (fees) we need to hold with us. That will also help than using drug at all time P5.

I want to say that you should go to radio station, advertise it. Then just go to all these pharmacists and advertise it.

### Barriers and facilitators to the implementation process

HCPs explained that major barriers to implementing the JIGSAW-A model of care were linked to the healthcare system structure and workflow (including lack of agreed and established guidelines to underpin care and reduce variation across settings, lack of truly collaborative/multidisciplinary care, conventional referral systems that are over-reliant on doctors in secondary/tertiary care). Other key barriers were resources and time constraints. High patient caseload was a barrier relating to time constraints and was cited by most participating community pharmacists and some other HCPs. Under current schedules, with so many patients expected to be seen each day, there seems to be too little time to deliver all of the elements of the JIGSAW-A model of care to all patients in community pharmacy settings. The resources provided as part of the JIGSAW-A study were limited, and without sustained sponsorship and/or adoption by the healthcare system, it would be difficult to continue to optimise patient empowerment for self-management through education and provision of these information resources.

From the patient perspective, finances and lack of community/primary care-based services to continue to support self-management are major barriers. Unaffordable consultation and care costs (direct out-of-pocket expenses or via health insurance schemes that do not cover physiotherapy services) make evidence-informed care that includes physiotherapy-led exercises an unaffordable ‘luxury’. Many patients expressed their need for care for coexisting LTCs and symptoms alongside improving care for joint pain and osteoarthritis.

Including the other problems I mentioned also… You know you are just explaining this one to me, I only glanced through the book without understanding. It is until now that you are explaining to me that I am getting it. P5

HCPs expressed that the JIGSAW-A resources increased their and patients’ insight and broad experience of self-management of osteoarthritis as an LTC (ie, that will not be cured by medicines). The resources were also perceived to enhance consultations and patients’ potential to take care of their own health. HCPs further highlighted that for the model of care to be effective and have a meaningful impact nationally, widespread HCP training will be needed. Adaptation of the JIGSAW-A guidebooks in a more engaging visual format (ie, short video) was a desire and perceived facilitator from both patients’ and HCPs’ perspective.

### Phase 4: stakeholder codesign of recommendations for wider implementation

**Figure 4:**
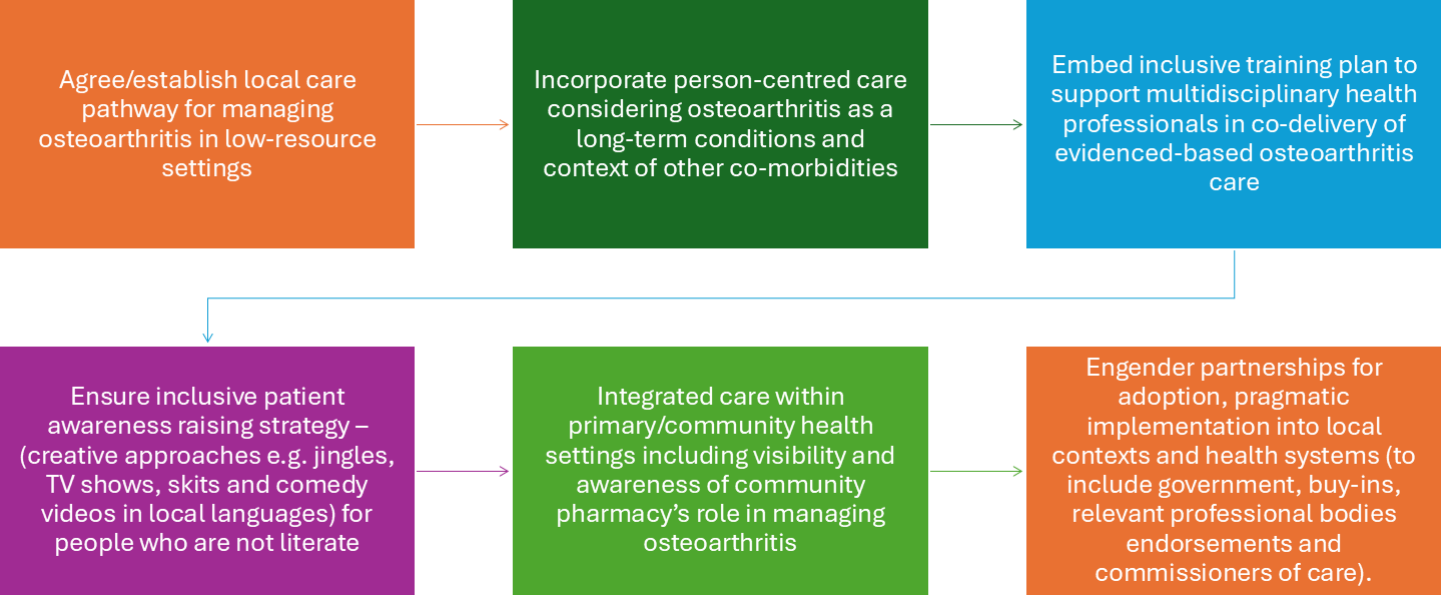
Stakeholder recommendation for wider implementation of JIGSAW-A.

Following pilot implementation and evaluation in real-world settings (in phase 3), refinement and optimisation of JIGSAW-A resources were guided by stakeholders. Three CoP workshops were held to define and prioritise recommendations for further scale-out and wider implementation of evidence-based, integrated care for osteoarthritis in Africa. The first was focused on a review of feasibility study data to agree on changes/amendments to the JIGSAW-A resources using an adapted Nominal Group Technique.^18^ Subsequent CoP workshops generated draft recommendations and prioritised strategies for wider scale-out and implementation of evidence-informed osteoarthritis care in West Africa. Strategic priorities and agreed recommendations are presented in figure 4.

## Discussion

This feasibility study incorporated stakeholder inputs, patient and public involvement, and leveraged previous learning and expertise from a similar successful osteoarthritis research and implementation programme in Europe (ie, JIGSAW-E). The diverse nature of the practices participating in the study, in terms of staffing, patient population size, urbanisation and deprivation, suggests that the results should have transferability to other settings in Nigeria and West Africa. Our findings show that a guideline-informed model of care can progress the understanding and management of osteoarthritis among HCPs and patients and is feasible to implement in overburdened health systems and low-resource settings. However, addressing key barriers and facilitators would be necessary, and the impact of the JIGSAW-A model of care and findings presented in this study needs to be tested for effectiveness in a large, adequately powered clinical trial.

Quantitative and qualitative evaluation of the implementation process and outcomes of guideline-informed osteoarthritis management programmes is expanding globally,^8 19 20^ including an emerging body of literature examining osteoarthritis management in community pharmacies,^21^ but there is a paucity of research focused on how people living in Africa with osteoarthritis and joint pain will perceive such programmes. To our knowledge, this is the first study to focus on the acceptability of written information resources on osteoarthritis and joint pain from the patient’s perspective in West Africa. We did find that information was an expressed need, and written resource was appreciated; however, Africans appear to want information in more engaging formats, such as videos and posters in addition to written information. These findings can help inform the population-wide implementation of this model of care in other regions with similar contexts.

Aside from limited resources and access to physiotherapy services, our study highlights poor integration of multidisciplinary HCPs and services across healthcare settings as a major barrier to effective implementation of guideline-informed care for osteoarthritis. This is similar to findings of previous literature in the field^7 8 19 20^ and in higher-resource settings. Professional roles, boundaries and patient expectations were also key, as community pharmacists in this study routinely supplied topical analgesics for most of their patients instead of oral analgesics and strong pain killers, which were usual practice prior to JIGSAW-A. Our study makes an important contribution to the literature on osteoarthritis care and acceptability from a low- and middle-income country (LMIC) perspective. The current study was a feasibility pilot project in Nigeria, with a view to extend learning and scale out JIGSAW-A innovations within Nigeria and to other countries in West Africa.

Limitations of the study include that the analyses did not account for repeated visits by patients during the pilot implementation period, due to limited resources for the study. However, the study was set up to answer questions about the feasibility of implementation in community settings and with community pharmacies. While being the usual first point of call for patients, and a great point to signpost, we have learnt that fidelity to a full JIGSAW-A model of care may not be best placed in a fast-paced business environment without undue pressure on patients and community pharmacies. We did find that JIGSAW-A worked mostly well at the community health centre. Findings of better compliance with osteoarthritis assessment protocol and physiotherapy-led exercises in JIGSAW-A in such sites were not surprising, given the expertise and close professional connections with osteoarthritis and joint as a condition. The JIGSAW-A training in such instances served to up-skill professionals and encourage better compliance to guideline-informed care.

Though relatively simple to use and enhances health professional behaviour/practice in line with best evidence for osteoarthritis, our point-of-care quality indicator recording template was not well used. Within context, routine recording of care does not usually take place in community pharmacies in West Africa/LMICs’ primary care. Future research aimed at maximising the benefit from the evidence-based osteoarthritis implementation programme should include a focus on wider uptake of routine electronic data collection, which will aid audits and support health professional behaviour changes that can reduce variations in practice and thereby improve patient-level outcomes.

### Implications for future policy and practice

This study responds to global policy, research and public health priorities by enabling provision of accessible and high-quality patient explanation packages and osteoarthritis care support packages for community pharmacies and other HCPs. Participants and stakeholders further recommended more visual, expressive arts and other creative means of giving education and advice for the management of osteoarthritis. This is particularly important in Africa where a high proportion of people living with osteoarthritis may have low health literacy.

People with osteoarthritis commonly have other LTCs, such as cardiovascular diseases, hypertension, peptic ulcers, diabetes, anxiety and depression.^22 23^ Future research needs to provide self-management programmes for osteoarthritis in the context of other coexisting LTCs. Equipping community pharmacies and other HCPs in primary care to support evidence-based osteoarthritis care as an LTC will improve person-centred care, and potential risks and adverse effects associated with self-medication misuse and drug interactions^9 10^ can be mitigated. This will likely have a significant impact at population levels.

Although the team promoted increased confidence in trustworthy services, a major barrier to the implementation of the JIGSAW-A model of care with heavy reliance on community pharmacies was time and the potential loss of income from the promotion of non-pharmacological approaches to osteoarthritis management. For effectiveness, evidence-based care programmes for osteoarthritis such as JIGSAW-A should also be established in community health centres with links to local networks of community support groups promoting supported self-management. Such an implementation approach is likely to enhance efficient care pathways and effective multidisciplinary team approach to the management of osteoarthritis, optimising the use of public and individual resources.

## Conclusions

JIGSAW-A pilot is to our knowledge the first example of the implementation of an osteoarthritis management programme in primary or community care settings in West Africa. Our feasibility study showed that community pharmacies in low-resource settings can be supported to deliver aspects of evidence-informed osteoarthritis management as a first point of call. However, given fast-paced, community pharmacy settings, well-contextualised care, consistent with international evidence-based guideline recommendations, may be better placed in community healthcare centres. In addition to more adequately powered research evidence, the commitment of care commissioners and collective partnership of stakeholders are now needed to establish effectiveness and support the translation of evidence into practice in these settings. This will provide people with osteoarthritis better quality of life and reduce socioeconomic inequalities associated with living with osteoarthritis as an LTC.

## Supplementary material

10.1136/bmjgh-2024-018714 online supplemental file 1

10.1136/bmjgh-2024-018714 online supplemental appendix 1

## Data Availability

All data relevant to the study are included in the article or uploaded as supplementary information.
