# Supplementary material for: Process and feasibility of implementing guideline recommendations for the care of osteoarthritis in West Africa
Source: BMJ Glob Health. 2025 Jun 19;10(6):e018714. doi: 10.1136/bmjgh-2024-018714 (PMC12182005; doi:10.1136/bmjgh-2024-018714)
Supplement: online supplemental file 1 [file bmjgh-10-6-s001.docx]

**Supplemental Table 1. JIGSAW-A IMPLEMENTATION AND EVALUATION (USING Re-AIM Framework)**

| **RE-AIM Domains** | **Concept operationalised** | **Outcome Target Level(s)** | **Measures/data source** | **Sample Quotes** |
| --- | --- | --- | --- | --- |
| Reach | HCPs and participating sites  Absolute number, proportion and representativeness of >45yrs with OA that participates in JIGSAW-A | Provider level (HCPs)  Individual | 1. recruitment data with participation rate 2. representativeness of HCPs and people living with OA participating 3. JIGSAW-A resources/dissemination products use/uptake – HCPs consultation logs and interviews | “First priority now and how we have used the guidebooks is for patient education” HCP7 |
| Effectiveness | Reflective of the success of JIGSAW-A in improving quality of care for people living with OA | Provider level (HCPs)  Individual | 1. Impact on HCP practice – interviews 2. Patient self-reported Quality Indicators of care for OA (% achieved) 3. Patient perception of JIGSAW-A program/resources and self-management - patient feedback/interviews | “I’m more conscious of the fact that we you don't need X-ray to diagnose osteoarthritis” HCP1  “So upon thorough examination and then I'm satisfied that OK, this patient can do well on exercise and diet like improving their way of life instead of just medications. So now I recommend exercise, diet, proper diet to my patients and I also illustrate to them how they can exercise, and many of them have come back with good feedback that is actually helpful” HCP2  “most of the times, the orientation or let me say the mentality that most of them have before is that the best solution or the only way to their problem is either to be on analgesic for a very long time or get a new support or any form of support. And ever since we've started introducing this guideline to them, we've been having a very, very positive result and the rate at which some of them even consume analgesic is reduced compared to the way it was.” HCP7  “Yes, I do exercise, I also have what I use to massage the legs, I also have hot water bottle. Also, when I wake up in the morning, I have to raise the legs on the bed and I try to do most of them. They’re easing the pain gradually” P4  “Infact, the tablet I used to take, I may stay for few weeks to a month without taking it. I only do the exercise. Infact, if I want to go to xx, I will leave my car here and just troll and there and come back” P11  Referring to the guidebook: “It is all encompassing since it contains everything that is wrong with us. It is like they saw us before they wrote it”. P5 |
| Adoption | Proportion, and representativeness of HCPs who train and uses JIGSAW-A in practice.  Regulatory bodies interests & uptake of JIGSAW-A | Provider level (HCPs)  Organisations | 1. Information from training and site visits. 2. HCPs interviews 3. Organisational engagements | “before the JIGSAW training, I don't really emphasize about their diets, or exercise. Yes, because it was not a concern. So basically, I recommended medications and supplements. I recommend gels or analgesic sprays” HCP2  “Our priorities: educating patients about their condition, importance of self-care, importance of lifestyle changes which include weight management and importance of exercise aligns with the JIGSAW A model of care. It aligns with that approach too, because I feel like that's basically what the JIGSAW-A is about; Self-management, weight management exercises. Yes, that's what I think we are doing”  “Actually, what we do before JIGSAW is that when they complain like that, before we commence any treatment, we usually refer them to the lab and to run scans. Which JIGSAW has made us see that it is not necessary to allow the patients go through that kind of stress before the person can be diagnosed of having osteoarthritis” HCP7  “Well, before JIGSAW started, I'm not really a fan of you do so much of exercise to support your joint. So I believe so much in nutritional supplement Condroitine, glucosamine, MSN and other ones that works. But ever since I had training with JIGSAW, I believe more in exercising those joints rather than you taking so much of supplement. Even though I still give supplements. But at least that goes a long way, alongside with the JIGSAW recommendation” HCP1. |
| Implementation | HCPs fidelity to JIGSAW-A+ model of care/protocol.  High priority on context specific analysis from qualitative data. | Provider level (HCPs) | 1. Consistency of implementing JIGSAW-A+ as intended, adaptations made during delivery assessed via site visits aided 2. Notes on local adaption at implementing sites assessed during site visits 3. Interviews | “If there is a need to I refer, I refer them to other healthcare professionals” HCP1  “The training has helped me to be mindful of the terms, the words I use, so as not to create any negative psychological impact on these patients as regarding their pain” HCP7  “There’s need for more collaboration. Everybody has their own professional jurisdiction and all of us need to look at how best can we help patients. So I would say that here, if JIGSAW A can bring all of us together, why not? For myself I would say that yes, it opened my eyes so that I can actually work more with physiotherapists”HCP6  “But in most cases after we have a very short discussion guide with the patient, we give them the material that is given to us, which is very, very informative and it's very, very precise, straightforward. So most of the patients we give it to them to still go and read and come back and some of them will come back to give us the feedback”. HCP3  “And also, I try to give them positive words as much as possible. I don't say death sentence, like it won’t be repairable again. So you're conscious of the kind of language being used” CP2  Yeah, it's very essential for health professionals to collaborate on the management of OA and that will really help us in Nigeria. We still have a long way to go, but at least we can start from like networks -collaboration between health professionals in the proper management of patients with OA. HCP 8 |
| Maintenance & Sustainability | Individual patients’ motivation for self-management and HCPs practice maintained/continues to be informed by JIGSAW-A in the longer term. | Individual  Provider level (HCPs) | 1. Individual level maintenance: continued interest in and incorporation of self-management (interviews) 2. HCP practices post-implementation (interviews) 3. Broader stakeholder engagement/ additional JIGSAW-A training delivered/requested | “JIGSAW A. is more of a physiotherapy tailored kind of something to be very honest. I'm a pharmacist so I would have my own different professional perspective and physiotherapy deal more with physical therapy and how that can be helpful. I may be wrong but pharmacists, do both pharmacological and pharmacological ways to help our clients. So I would think if JIGSAW A and pharmacists work together, it could work because we need each other” HCP8.  Readability and Literacy: “Africans are not used to reading : The feedback has been good, though we still have some of them that Nigerian mentality? No, not only Nigerians. You know, Africans. Just like they use to say, which is not 100% true, that the best way to kill A to kill a black man is to put it into writing. I'm just joking, but some of them still find it difficult to read. Not because they cannot read, actually, but some of them are still very, very awkward in terms of even if you give them the Yoruba version to read, they will still come back and still ask you that they still don't understand that you should put them through. And you later discovered that they didn't even read it the way they're supposed to. But for people that read it, digest it very well, they still come back to say thank you and that I even gave this one you gave me to another person, which is very, very good”. HCP1  The food palette…that if I am to follow them I will use all my salary on feeding. You have you go and look at the realities of our nutrition. So you can make realistic recommendations to the people. Medical services are too expensive so many people will rather self-medicate than come to a doctor. I think that is the issue and I don't know how your organization can step in. Even self-medications are getting expensive so many people are resulting to herbs. The problem now is some people are lucky it works for them. But it gets to a time that it will not work, that is why we have an increase in liver problems” P5  “especially these fruits... It has been useful to me alot. I buy watermelon, orange and other fruits. Before, I don't use to take fruits. But now, I take fruits and vegetables very well. But the cost implications! If you want to buy little banana now, they can say 200 or 300 naira. Then the government, I don't know what to say about them. How they will decrease the inflation?” P3  “Yes, the guidebook was easy to read but I thought they should have monitored me and ask us to come for regular session” P2 |
| **Barriers & Facilitators** | **Sample Quotes** | | | |
| Barriers | “I believe in physiotherapist, if there is an opportunity or privilege, they can go for physiotherapy but for most patients, such luxury is not available” HCP7  “we can know what exactly is wrong with them because it is due to their poverty level that they didn't go for physiotherapy that is number one. Secondly, I want them to create awareness, for instance where I am now we have about 32 villages around and I don't have the opportunity to see them all but if some other people can be trained to explain to other people, that will go a long way” N1  “what I think about the diet we ought to be taking, right? Do you think we can follow this thing in Nigeria? It is really good but the monetary aspect is not easy. It is not realistic!”  “It was mentioned to me but I didn't get a copy”  “They just gave me the book yesterday that I should look at and this Is what I should buy. They showed me the low and high quality. But I don't have money. But they told me this one is the best one and I went for it. I still owe him money for this for him to give me better one. P3  “I have had several tests but I believe the ones I did at the physiotherapist place is the only solution to my problem. Since the other week at XXX physiotherapy, and I observed there were changes, but I didn't have money to go back to them. P9 | | | |
| Facilitators | “And then also I look forward to when there will be like osteoarthritis group like maybe support group for the osteoarthritis patients around here like they just have a support group where they come together to discuss about what they're going through and also there will be professionals to attend to them, maybe some of the facilities for exercising or maybe tell them about their health, their nutrition, what they're supposed to eat and what they're not supposed to eat”. HCP 7  “even if you give them the Yoruba version to read, they will still come back and still ask you that they still don't understand that you should put them through. And you later discovered that they didn't even read it the way they're supposed to. I say that we should make it in an audio form, and something catching in form of a poster that will give the basic information at once and that will be fine”  “Well, it's just a book, so maybe videos like the exercise, demonstrating to patients how it's been done or maybe like a JIGSAW support group that will be like something that has to be online, like one-on-one conversation”. HCP2  “So I think the training has gone a long way but it has to be something that is sustaining”. HCP3 | | | |

Supplementary Table 1. Summary of site data (extracted from consultation logs and HCP self-reports)

| Site code |  |  | Demographics | | | | | | Assessment & management Plan | | | | | | | | | | | |
| --- | --- | --- | --- | --- | --- | --- | --- | --- | --- | --- | --- | --- | --- | --- | --- | --- | --- | --- | --- | --- |
|  | *OA patients per day (average)* | *Total no of Patient consultations (JIGSAW-A) 3months* | *%Chronic* | *Gender ratio (M:F)^~^* | *% Obese/ Overweight* | *% other LTCs* | | | Assessment as per protocol | Education on OA & Joint Pain (inc. JIGSAW-A booklet) | Advise: Self-Management | Prescribed pain medications | Prescribed topical analgesics | Imaging/ Diagnostic Tests | Recommend Exercise | Prescribed equipment (e.g. walking aid) | Referral - Physiotherapy | Referral - Orthopaedic /Others | Red flags (JIGSAW-A algorithm) |  |
| *Urban* |  |  |  |  |  |  | | |  |  |  |  |  |  |  |  |  |  |  |  |
|  |  |  |  |  |  | Hypertension | *Diabetes* | Others |  |  |  |  |  |  |  |  |  |  |  |  |
| A1001 | 13 | 43 | 75 | 12:5 | 44 | 84 | 30 | 30 | 5 | 43 | 43 | 23 | 43 | 28 | 43 | 3 | 0 | 14 | 5 |  |
| A1002 | 8 | 23 | 100 | 1:2 | 57 | 13 | 52 | 9 | 0 | 23 | 15 | 23 | 23 | 0 | 17 | 1 | 0 | 0 | 0 |  |
| A1006 | 8 | 35 | 48 | 1:4 | 29 | 31 | 17 | 14 | 3 | 35 | 34 | 14 | 35 | 4 | 34 | 2 | 13 | 1 | 3 |  |
| A1007 | 10 | 39 | 97 | 1 | 74 | 59 | 59 | 21 | 0 | 39 | 39 | 39 | 39 | 13 | 29 | 1 | 24 | 13 | 0 |  |
| A2001 | 5 | 60 | - | 1 | 43 | 48 | 42 | 18 | 60 | 60 | 60 | 7 | 60 | 9 | 60 | 7 | 0 | 7 | 60 |  |
| A3001 | 11 | 44 | 54 | 5:8 | 75 | 68 | 34 | 20 | 44 | 44 | 44 | 13 | 5 | 7 | 41 | 5 | 0 | 4 | 44 |  |
| Rural |  |  |  |  |  |  |  |  |  |  |  |  |  |  |  |  |  |  |  |  |
| A1003 | 4 | 51 | - | 11:3 | 57 | 49 | 45 | 12 | 10 | 51 | 51 | 35 | 51 | 7 | 42 | 3 | 2 | 5 | 10 |  |
| A1004 | 7 | 41 | ~85 | 4:7 | 76 | 56 | 61 | 10 | 0 | 41 | 41 | 41 | 41 | 11 | 26 | 1 | 22 | 15 | 0 |  |
| A1005 | 6 | 33 | - | 13:4 | 55 | 42 | 27 | 18 | 5 | 33 | 33 | 7 | 33 | 3 | 30 | 0 | 7 | 3 | 5 |  |
| Total | 72 | 369 |  |  | 510 | 450 | 367 | 152 | 127 | 369 | 360 | 202 | 330 | 82 | 322 | 23 | 68 | 62 | 127 |  |

- not reported, ~ estimated
