# Supplementary material for: Process and feasibility of implementing guideline recommendations for the care of osteoarthritis in West Africa
Source: BMJ Glob Health. 2025 Jun 19;10(6):e018714. doi: 10.1136/bmjgh-2024-018714 (PMC12182005; doi:10.1136/bmjgh-2024-018714)
Supplement: online supplemental appendix 1 [file bmjgh-10-6-s002.pdf]

Training Outline

Module 1: Introduction: expectations of the training, background and findings from JIGSAW-A study

Module 2: The burden and impact of joint pain and osteoarthritis in people aged 45 years and over

- Global, Africa, Nigeria – scale of impact and burden
- Everyday living with joint pain (Elizabeth's story - video)

Module 3: What is joint pain and osteoarthritis and how to explain it well

Module 4: Delivering the JIGSAW-A model of care and the JIGSAW-A tools with case examples

- Community based care pathway for osteoarthritis – JIGSAW-A MODEL
- Helping people manage their joint pain and osteoarthritis
- Broaching weight within a consultation
  - Cultural sensitivities

Module 5: The unique role of community pharmacists, Physiotherapists and Doctors in joint pain and osteoarthritis care

- History taking, joint examination (observation)
- Ruling out red flags (algorithm)
- Excessive/ Routine Investigations and Imaging
- Autonomy of practice, Task shifting and Multidisciplinary team working

Module 6: Participating in the JIGSAW-A feasibility study: full study process

JIGSAW-A model care consultations – Decision Algorithm

Serious pathology	Examples	Indications
Infection	Septic arthritis<br>Osteomyelitis	Redness, swelling, heat.<br>Fever, sweats, chills.<br>History/indications of immunosuppression.<br>Difficulty weight-bearing.
Inflammatory	Rheumatoid arthritis<br>Polymyalgia rheumatica<br>Crystal arthritis e.g., gout	Pain that worsens at rest.<br>Persistent joint swelling and tenderness.<br>Joint warmth and/or erythema.<br>Morning stiffness ≥30 minutes.<br>Unexplained weight loss.
Fracture***	Osteoporotic fracture<br>Traumatic fracture	Sudden onset of pain.<br>Incidence of trauma.<br>History of osteoporotic fracture.<br>Rapid worsening of symptoms or deformity.
Comorbid LTCs?<br>Others	Diabetes, Hypertension<br>Recent surgery***<br>TB, steroids use, Pneumonia	immunosuppression

Purchase or prescription about peripheral joint problem/ pain/ OA (knee, hand, hip, foot)?

- Patient 45 years or over **and**
- have activity-related joint pain
- have either no morning joint-related stiffness or morning stiffness that lasts no longer than 30mins.

Positive criteria?

YES

- Ask about type and amount of pain?
- Regular analgesia?
- Ask about how joint pain is currently being managed and how they are coping
- Other comorbidities (hypertension & Diabetes)?
- Red flags protocol

JIGSAW-A model match?

Less complex needs

JIGSAW-A toolkit

- Offer the JIGSAW-A OA guidebook.
- Offer simple analgesic advice (topical NSAIDs, simple non-pharmacological pain relief (e.g., hot/ cold).
- Encourage exercise and activity.
- Encourage weight loss if applicable.
- Signpost to support groups
- Signpost to Physiotherapist

NO

More complex needs

Cascade for Licensed Community Pharmacist consultation /
Refer to Physiotherapist/
Refer to Physician for medical review

Referral for specialist care if applicable:
- Joint replacement - Orthopeadics
- Occupational therapists
- Complement with JIGSAW-A toolkit

© JIGSAW-Africa 2023
